# Monte Carlo simulations of out‐of‐field skin dose due to spiralling contaminant electrons in a perpendicular magnetic field

**DOI:** 10.1002/mp.13392

**Published:** 2019-02-14

**Authors:** Victor N. Malkov, Sara L. Hackett, Bram van Asselen, Bas W. Raaymakers, Jochem W. H. Wolthaus

**Affiliations:** ^1^ Department of Radiotherapy University Medical Center Utrecht Utrecht The Netherlands

**Keywords:** EGSnrc, magnetic field, Monte Carlo, MRgRT, out‐of‐field dose

## Abstract

**Purpose:**

The purpose of this study was to evaluate the potential skin dose toxicity contribution of spiralling contaminant electrons (SCE) generated in the air in an MR‐linac with a 0.35 or 1.5 T magnetic field using the EGSnrc Monte Carlo (MC) code. Comparisons to experimental results at 1.5 T are also performed.

**Methods:**

An Elekta generated phase space file for the Unity MR‐linac is used in conjunction with the EGSnrc enhanced electric and magnetic field transport macros to simulate surface dose profiles and depth‐dose curves in panels located 5 cm away from the beam edge and positioned either parallel or perpendicular to the magnetic field. Electrons generated in the air will spiral along the magnetic field lines, and though surface doses within the field will be reduced, the electrons can contribute to out‐of‐field surface doses.

**Results:**

Surface dose profiles showed good agreement with experimental findings and the maximum simulated doses at surfaces perpendicular to the magnetic field were 3.77 ± 0.01% and 3.55 ± 0.01% for 1.5 and 0.35 T. These results are expressed as a percentage of the maximum dose to water delivered by the photon beam. The surface dose variations in the out‐of‐field region converge to the 0 T doses within the first 0.5 cm of material. An asymmetry in the dose distribution in surfaces positioned on either side of the photon beam and aligned parallel to the magnetic field is determined to be due to the magnetic field directing electrons deeper into, or localizing them to the surface of, the measurement panel.

**Conclusions:**

These results confirm the SCE dose contribution in surfaces perpendicular to the magnetic field and show these doses to be of the order of a few percentage of the maximum dose to water of the beam. Good agreement in the dose profiles is seen in comparisons between the MC simulations and experimental work. The effect is apparent in 0.35 and 1.5 T magnetic fields and dissipates within the first few millimeters of material. It should be noted that only SCEs from beam anteriorly incident on the patient will influence the patient surface dose, and the use of beams incident over different angles will reduce the dose to any particular patient surface.

## 1 Introduction

Magnetic resonance‐guided radiation therapy (MRgRT) provides an exciting prospect of real‐time tumor tracking and adaptive radiation therapy.^1^, ^2^, ^3^ Implemented and developing systems consist of magnetic resonance imaging machines with magnetic fields ranging from 0.35 to 1.5 T and radiation delivery is achieved using Co‐60 or linac‐based photon sources. Prototypes of the Australian MR‐linac^4^ and the MagnetTx Aurora‐RT system^5^ are capable of delivering radiation beams parallel to the magnetic field. The Viewray MRIdian^6^ (Co‐60 or linac) and the Elektra MR‐linac^7^, ^8^ employ a gantry system in which the incoming radiation beam is always perpendicular to the magnetic field.

The impact of the magnetic field on electron trajectories, and consequently on dose distributions,^9^, ^10^ has been well established. A magnetic field orthogonal to the incoming radiation beam induces several effects on the dose distribution.^11^, ^12^, ^13^ One of these is the electron return effect (ERE), which occurs in regions of sharp density changes and is responsible for an increase in the interface surface dose. Further effects include a dose shift, toward the direction perpendicular to both the beam and magnetic field, and an increase in the maximum dose deposition, due to the electron energy being deposited closer to the point of photon interaction. Parallel magnetic and photon field setups have been shown to have an increase in surface dose due to magnetic containment of scatter electrons from the linac head and irradiated air.^4^, ^14^, ^15^, ^16^ Conversely, a perpendicular magnetic field sweeps contaminant electrons out of the primary field and induces a reduction in doses to surfaces along the direction of the photon field (although this reduction may be obscured by the ERE). These surface dose variations are relative to the no magnetic field cases in which contaminant and backscattered electrons would, for the most part, be able to scatter away from the incident beam.

Recently, Hackett et al.^17^ explored the out‐of‐field dose contribution due to spiralling contaminant electrons (SCE) in the Unity MR‐linac installed at the University Medical Center Utrecht. Electrons are generated in the air,^18^, ^19^ away from the linac head. Subsequently, these “airborne” electrons are swept out of the primary field and will continue along their trajectories as they spiral along the magnetic field lines. The magnetic field lines are oriented along the superior–inferior orientation of the patient. Therefore, potentially, these SCEs can strike the patient and induce surface dose contributions outside the radiation treatment field. Hackett et al. found that the surface dose, positioned at 0.13 mm depth, was 5.6% of the maximum dose‐to‐water deliverable by the photon beam, for a 10 × 10 ${\text{cm}}^{2}$ field size measured 5 cm away from the field edge and perpendicular to the magnetic field. Measurements in films placed parallel to the magnetic field showed a lower surface dose compared to the perpendicular measurements. This is due to the air‐generated electrons spiralling along the magnetic field lines and toward the surfaces perpendicular to the magnetic field. Furthermore, an asymmetry in the dose measurements was observed based on which side with respect to the beam the measurement panel parallel to the magnetic field was positioned.

Monte Carlo (MC) simulations can often yield additional physical insight into dosimetry measurements. In this study, we provide detailed EGSnrc^20^ simulation results of the SCE out‐of‐field doses with supported explanation of the observed effects. The simulations are set up to reflect the 1.5 T measurements of Hackett et al.^17^ In addition to 1.5 T, the SCE effect is explored in 0.35 T and also compared to 0 T out‐of‐field doses.

## 2 Materials and methods

The egs_chamber^21^ application of the EGSnrc Monte Carlo code system^20^ is used for all presented simulations. egs_chamber is a *c++*‐based application which permits general dose calculations using the Fortran‐based EGSnrc transport physics in a range of predefined geometrical structures. The influence of the magnetic field is included using the recently implemented and validated enhanced electric and magnetic field macros.^22^ The specialized boundary crossing algorithm, scaled $\delta_{u}$, and adaptive integration algorithms are employed for the magnetic field transport. The EM ESTEPE parameter, equivalent to $\delta_{u}$, which limits the step size in PRESTA‐II electron transport to allow for a maximum fractional change in the direction of motion due to the magnetic field, is set to 0.2 as recommended in Malkov and Rogers.^22^ The robustness of the results to variations in this parameter is discussed in Section 3.D. Electron (ECUT) and photon (PCUT) total energy cutoffs were set to 521 and 10 keV, respectively. The full simulation parameters are listed in Table 1. Simulation parameters, ECUT and EM ESTEPE, were varied only in the simulations of Section 3. D. The only variance reduction technique used was photon cross‐section enhancement (CSE), for which the enhancement factor was set to 8 or below based on individual simulation optimization. CSE was not used for any egs_chamber calculations of the phase‐spaces at the surfaces of the water phantom.

**Table 1:** EGSnrc simulation parameters

Parameter	Value
Photon cross sections	XCOM
Compton cross sections	EGSnrc default data
Pair cross sections	Bethe–Heitler
Pair angular sampling	Simple
Triplet production	Off
Bound Compton scattering	Norej
Radiative Compton corrections	Off
Rayleigh scattering	Off
Atomic relaxations	On
Photoelectron angular sampling	On
Photonuclear attenuation	Off
Photonuclear cross sections	EGSnrc default data
Brems cross section	NIST
Brems angular sampling	Koch–Motz
Spin effects	On
Electron impact ionization	Off
Global smax	1e10 cm
ESTEPE	0.25
Ximax	0.5
Boundary crossing algorithm	Exact
Skin depth for BCA	3
Magnetic field^a^	0, −1.5,0
EM ESTEPE	0.2

The magnetic field is shown for only for the 1.5 T magnetic field case.

The particle source used was the latest phase space file (provided by Elekta) for the 7 MV Elekta Unity MR‐linac. The phase space is scored at 129.5 cm away from the particle source and defines field size at isocenter (located 143.5 cm away from the particle source). The majority of simulations are performed using a 10 cm × 10 cm field size, but a 2 cm × 2 cm and 22 cm × 22 cm phase space files are also utilized to assess field size effects. Both electrons and photons are included in the phase space file; however, the electrons were not included for magnetic field (0.35 and 1.5 T) simulations since these electrons spiral along the magnetic field lines. Furthermore, their radii of curvature in the magnetic field are too small to be able to reach the scoring regions used in this study. The exclusion of these electrons improved simulation efficiency, and test simulations comparing the influence of including or excluding the phase space electrons from the simulations show no difference on the results presented in this work.

### 2.A. Simulation setup

**Figure 1:**
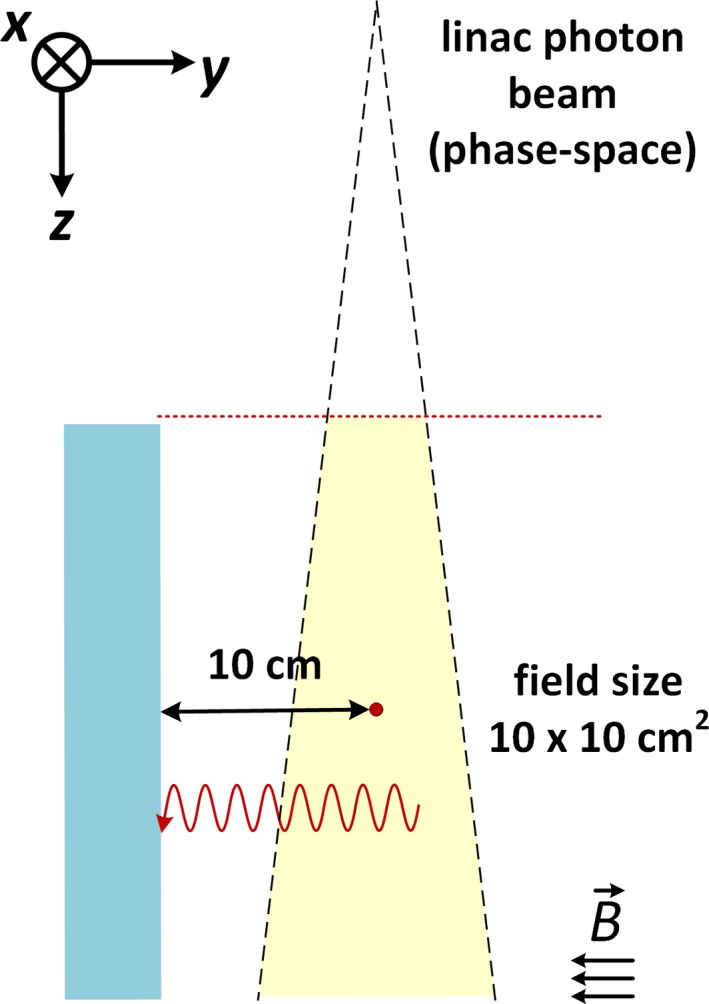
Schematic of the simulation setup with a 7 MV linac phase space source with the field size (10 cm × 10 cm shown) defined at isocenter (red dot). The dashed red line represents the scoring plane of the phase space file located 129.5 cm away from the source. A water panel with dimensions of 20 cm × 5 cm × 30 cm along the x, y, and z axes, respectively, is positioned 10 cm away from isocenter. The red sinusoidal line symbolizes a contaminant electron which would spiral along the magnetic field lines. [Color figure can be viewed at http://www.wileyonlinelibrary.com/]

A simple schematic of the simulation geometry is shown in Fig. 1. The photon beam is transported entirely through a larger air phantom with a water panel with the surface center located 10 cm away from isocenter. The water phantom is 20 cm × 5 cm × 30 cm in the x, y, and z axes in Fig. 1. The IEC‐1217 convention for machine geometry is used. The magnetic field is always oriented along the negative y‐axis. This simulation configuration reflects the experimental setup of Hackett et al. in which a series of alternating EBT3 and solid water slabs are similarly positioned outside the main photon beam and with the surface centered on isocenter. The EBT3 films are roughly 0.27 mm thick overall with a central sensitive region of 0.028 mm thick.

**Figure 2:**
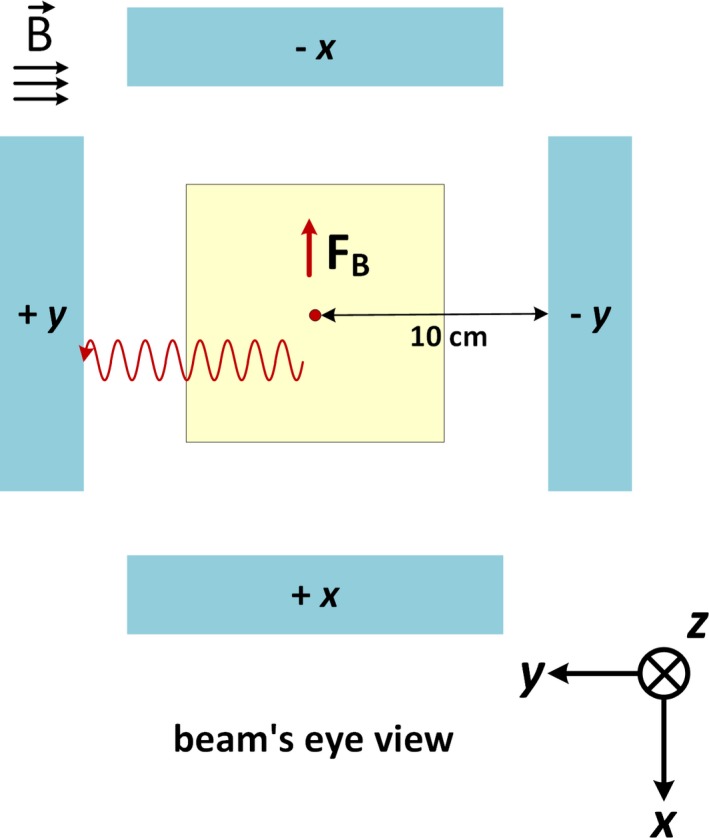
Beam’s eye view of the simulation setup demonstrating the four positions used for the scoring panels. Simulations were always performed with a single panel present to avoid interpanel interactions. The sinusoidal red line is a sample spiralling electron along the magnetic field, and the red arrow (marked as $F_{B}$) is the initial direction of the Lorentz force on an electron generated along the direction of the photon beam (into the page). [Color figure can be viewed at http://www.wileyonlinelibrary.com/]

Figure 2 shows the simulation setup from the beam’s eye view and demonstrates the four orientations of the measurement panels used in the simulations. Simulations were performed with only a single panel present at a time to avoid any streaming of backscattered electrons from one of the y panels to the other. Electrons generated in the air will spiral along the y‐axis oriented magnetic field, as shown by the sinusoidal red lines in Figs. 1 and 2. In addition to the SCE effect, there is a dose contribution in all the panel from scatter photons outside the primary field. These photons are included in the phase space file.

In all of the panels, a line profile along the direction perpendicular to the incoming photon beam is scored. The profile is scored in an 18 cm × 0.03 cm × 1 cm region in the x, y, and z directions in Fig. 1. The 18 cm long region is segmented into 72 regions (each 0.25 cm wide). The profile is scored at the surface of the water panel and at depths of 1.4, 2.3, and 4.0 mm into the panel to match the Hackett et al. measurements. The surface simulation is centered at 0.15 mm depth. Further depth‐dose curves are scored in a 1 × 1 ${\text{cm}}^{2}$ surface area region starting at the center of the surface of each of the panels. The voxels for the depth‐dose calculations are each 0.1 cm thick along the direction into the panel and away from isocenter. A magnetic field of either 0, 0.35, or 1.5 T is simulated. The 0.35 T magnetic field simulations are included to evaluate the potential effect of SCE dose contribution in an updated linac‐based Viewray MRIdian system, and currently, there are no experimental results available for comparison at this magnetic field. The Elekta phase space file is used for these 0.35 T simulations and should approximately reflect the magnitude of the effect in the linac MRIdian machine. All doses are reported as a percentage of the maximum deliverable dose, $D_{\max}$, by the beam in a 30 × 30 × 30 ${\text{cm}}^{3}$ water phantom with an SSD of 133.5 cm in each of the simulated magnetic fields. The maximum dose is determined by scoring the central axis depth‐dose curve in voxels with a 0.5 × 0.5 ${\text{cm}}^{2}$ surface area, and a thickness of 0.2 cm along the direction of the beam. Simulation uncertainties (k = 1) for individual voxels in all profiles are at most 0.35%, with the majority of simulations being below 0.2%. Uncertainties for maximum doses and side panel depth‐dose data are below 0.1%.

For the 1.5 T simulations and to match further experimental results from Hackett et al., the negative y panel surface profile calculations are repeated with the panel positioned at 15, 20, and 25 cm away from isocenter. To evaluate the SCE effect variations along the direction of the incoming beam, profiles from −10 cm to +10 cm away from isocenter along the z‐axis are determined for the four depths for the negative y panel. These profiles are calculated in voxels of 0.03 cm × 1 cm × 0.25 cm along the x, y, and z axes, respectively.

To assess the field size dependence of the SCE effect, simulations with the negative y panel and a 1.5 T magnetic field are performed with either a 2 cm × 2 cm or 22 cm × 22 cm phase space file. Profiles are scored along the x‐axis, and the dose is expressed as a percentage of the maximum dose, calculated for each field size. The negative y panel is positioned at 10, 15, or 25 cm away from the isocenter for the 2 cm × 2 cm field size, and at 15 and 25 cm away for the 22 cm × 22 cm simulations.

### 2.B. Experimental setup for 10 cm from isocenter

The experiments of Hackett et al.^17^ were repeated for the 10 cm panel position. Experiments were performed on the clinical Elekta Unity MR‐linac system at UMC‐Utrecht which corresponds the beam model of the phase space files used in the MC simulations. The experiments of Hackett et al.^17^ were made on a prototype MR‐linac system. Measurements were made with EBT3 model GAFCHROMIC films (Ashland Inc, Bridgewater, NJ) from the same lot (#06131702). Films were exposed as described in Hackett et al., with a film positioned on the surface of a solid water phantom, and three more films interspersed with layers of solid water 1 mm thick, so that the effective depths of the active layers of these films were 0.1, 1.4, 2.7 and 3.9 mm. The surfaces of films in contact with solid water were coated with water to eliminate air interfaces and thus prevent dose increases due to the electron return effect.^7^ The film stacks were positioned with the surface film located 5 cm from the geometric edge of a 10 cm × 10 cm field. Each film stack was exposed with a beam of 5000 MU (results are expressed as a percentage of 5000 cGy, the maximum deliverable dose in a water phantom).

The films were scanned approximately 48 h after exposure and calibrated as described in Hackett et al.^17^ The calibration curve was generated on the MR‐linac to account for any effect of the magnetic field on the response of the EBT3 films to radiation.^23^

### 2.C. Phase space scoring in the lateral (x) panels

For the 1.5 T case, in order to investigate the details of the effect of the magnetic field at the surfaces of the lateral x panels (surface parallel to the magnetic field), phase spaces are scored in a region with a size of 18 cm × 5 cm along the y and z axes, respectively. The phase spaces are scored either right at the surface of the phantom or at 0.5 cm deep. At the surface, only electrons generated by the photons from the source phase space in the air and entering the panels are scored. For the 0.5 cm phase space calculation, one simulation is performed for scoring electrons generated only in the first 0.5 cm of water the panel and travelling into (i.e., away from isocenter) the panel. The second phase space calculation at 0.5 cm depth is performed to score only electrons generated in the water beyond the first 0.5 cm and travelling out of the panel (towards isocenter). These phase space calculations allow for a comparison to be made between the particle fluences travelling into and out of the phantoms positioned on either side of the photon beam.

## 3 Results and discussion

### 3.A. 1.5 T results and comparison with experiment

**Figure 3:**
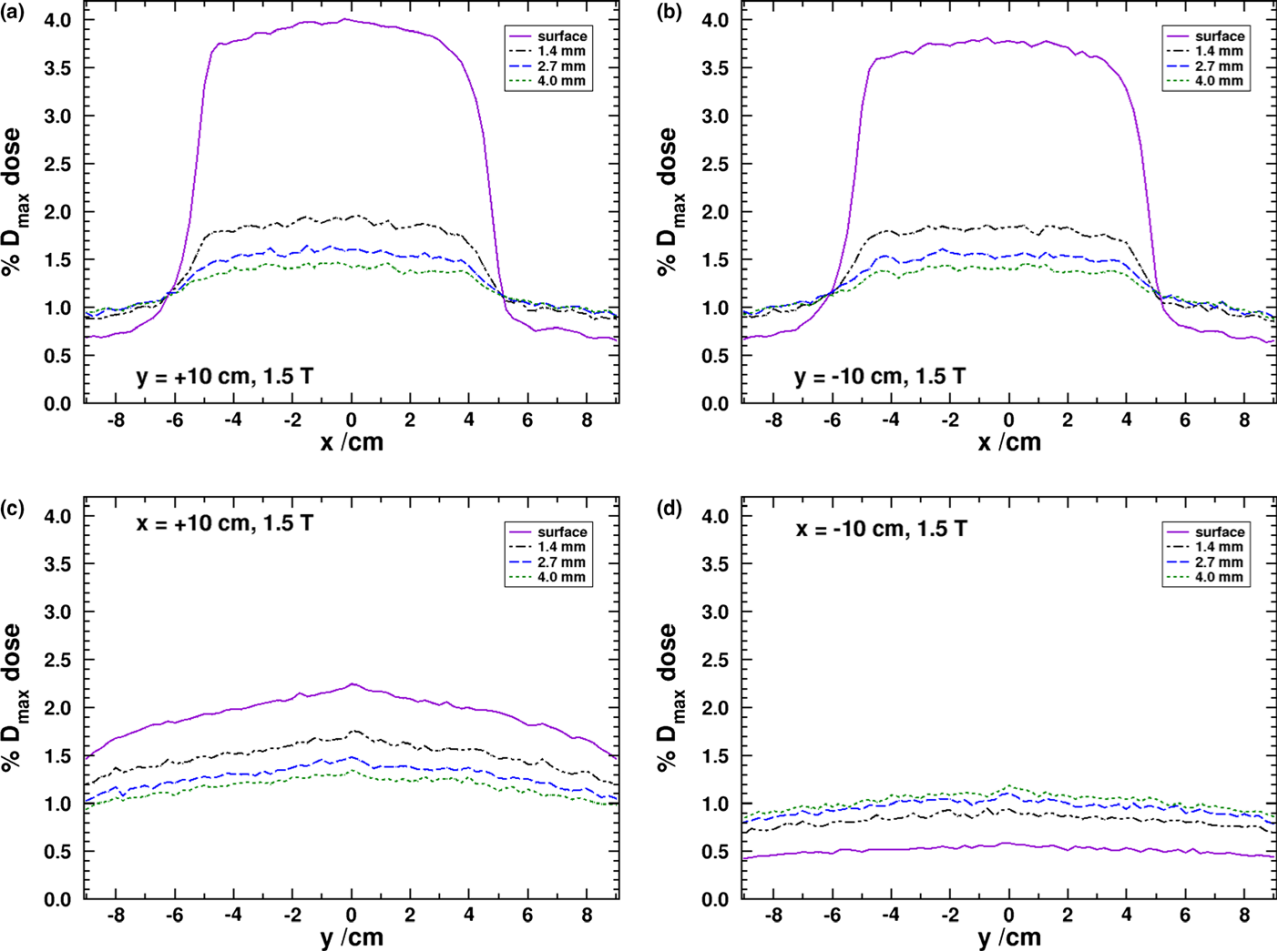
MC derived dose profiles at the surface and various depths in each of the panels shown in Fig. 2. [Color figure can be viewed at http://www.wileyonlinelibrary.com/]

In Fig. 3, the dose profiles for the four panel positions at multiple depths are shown for the 1.5 T simulations. As expected, the y panels observe a higher dose deposition than the x panels. Figures 3(a) and 3(b) demonstrate a similar response in the y panels since the SCEs have equal probability of scattering toward the positive or negative directions of the y‐axis; however, there is a slight difference between the surface doses of the two panels which could be due to an asymmetry in the directional distribution of photons in the phase space file. When a higher ECUT of 661 keV is used for the simulations, there is no statistically significant difference between the surface doses of the y panels, which would indicate that the contribution is coming from low‐energy electrons. The surface response, averaged in a 1 cm region centered at x = 0 cm, is found to be 3.99 ± 0.01% for the positive y panel and 3.77 ± 0.01% for the negative y panel. This is lower than the experimental result of Hackett et al. of 5.6 ± 0.2% and may arise from a difference between the phase space beam model and the experimental beam of the prototype MR‐linac.

**Figure 4:**
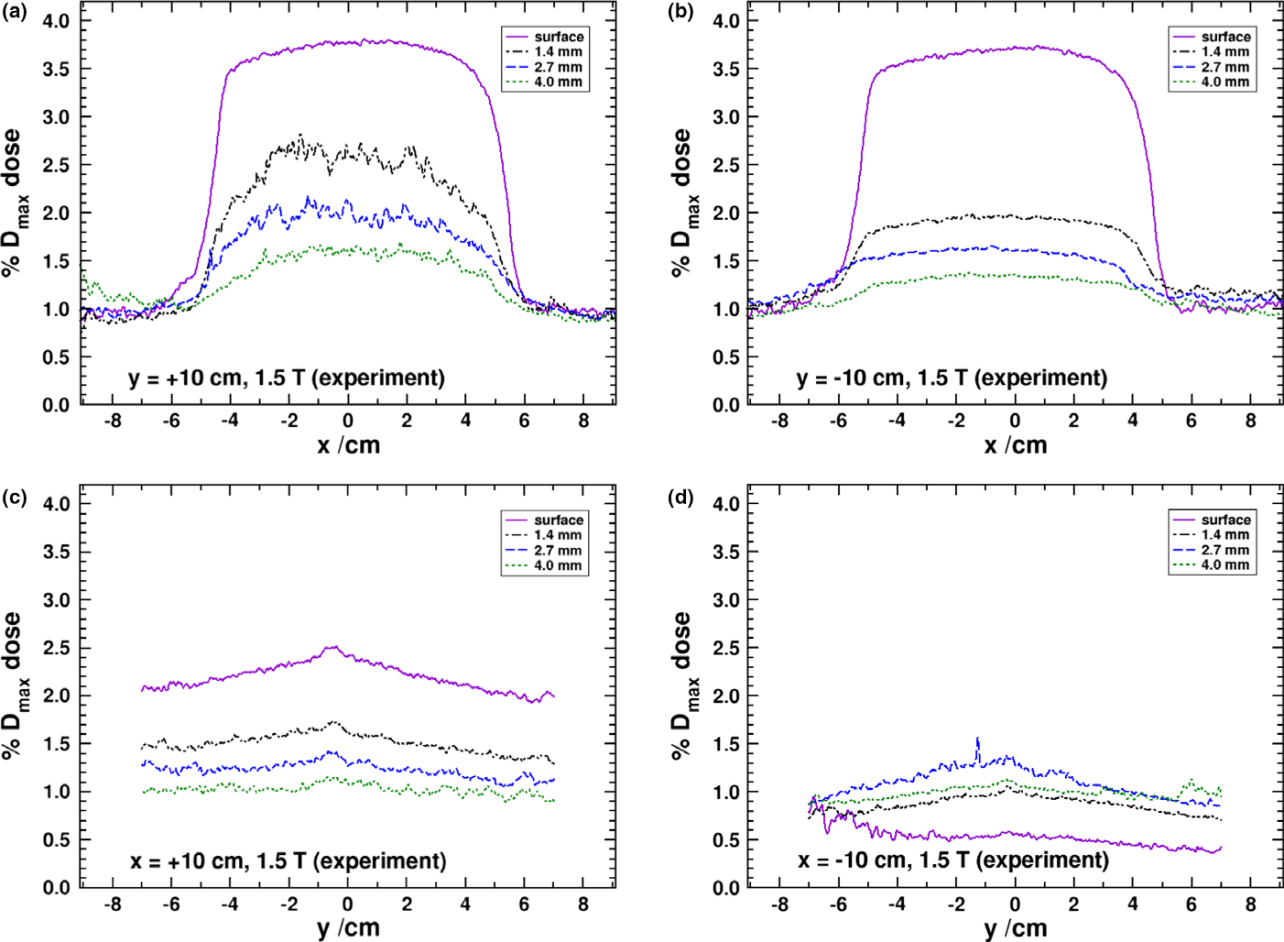
Experimental EBT‐3 film measurements with a 10 cm panel to isocenter distance. [Color figure can be viewed at http://www.wileyonlinelibrary.com/]

**Figure 5:**
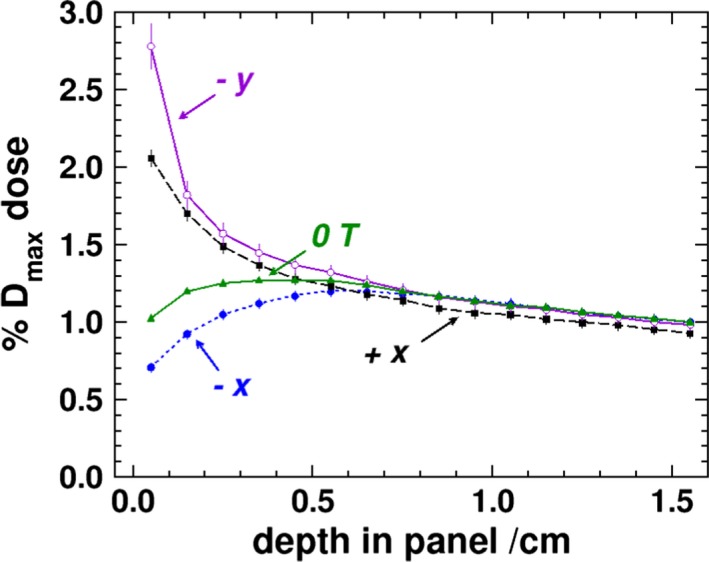
MC depth‐dose data starting from the center of the surface of the labeled panels. All results are for 1.5 T except for the labeled 0 T which are for the negative y panel (the 0 T simulations results are equivalent for all of the panels). Each point represents the dose averaged over a 1 mm thick region along the depth of the phantom. [Color figure can be viewed at http://www.wileyonlinelibrary.com/]

Due to these observed difference, the experimental results for panel positioned 10 cm away from isocenter have been repeated. These results are given in Fig. 4 and show an improved agreement between MC and experimental results. The positive y‐panel surface dose, averaged over a 1 cm wide region, is 3.77% as compared to 3.71% for the negative y panel (with an absolute experimental uncertainty of about 0.20%) which is similar to the asymmetric results seen in the MC simulation, though to a smaller difference in magnitude. At depth, the negative y‐panel doses are 2.0%, 1.6%, and 1.3% for the 1.4, 2.7, and 4.0 mm depths. The positive y‐panel results, though with increased noise, are 2.6%, 2.0%, and 1.6% for the same respective depths. For the same depths, the MC results are 1.84%, 1.54%, and 1.41% for the negative panel and 1.94%, 1.60%, and 1.44% (absolute uncertainty of 0.01%). The MC results are in good agreement with the depth results of the negative panel, while the experimental results of the positive panel are about 0.5% higher. In both the MC and experimental results, the region with increased dose in these panels is roughly 10 cm wide and reflects the width of the photon beam. There is an asymmetry about the x‐axis in the y‐panel results. The shift in the profile is due to the airborne electron being initially directed, on average, in the same direction as the photon beam. Since that direction is tangential to the helical path that the electrons follow and the influence of the magnetic field produces a rotation toward the negative x‐axis, the overall electron fluence becomes shifted in that direction. In the y‐panel profiles, it is clear that the dose drops off substantially within the first few millimeters of material. This is further demonstrated in the depth‐dose curves shown in Fig. 5 where it can be seen that deeper than 0.5 cm into the phantom doses in all of the panels converge close to the 0 T values.

The profiles of the x panels are given in Figs. 3(c) and 3(d), and, unlike the similarity of the positive and negative y panels, there is a distinct asymmetry, which was also observed in the experimental results of Hackett et al. and those in Fig. 4. In the positive x panel, the dose is highest at the surface, and in the negative x panel, there appears to be a buildup effect. This is further exemplified in the depth‐dose curves in Fig. 5. Due to the Lorentz force being initially directed toward the negative x‐axis (for electrons initially oriented along the primary beam’s direction), there is a preference for electrons generated in the air to strike the entry surface of the negative x panel. The phase space calculations at the surface reveal that there are roughly 2.7 as many electrons striking the negative x panel as the positive. It would be expected that due to this effect, the surface dose of the negative x panel to be higher than the positive one. However, it is the electrons generated by the scatter photons, from the phase space, that produce the overall difference seen in the profiles between the x‐axis panels. The phase space scoring at 0.5 cm into the phantom reveals that, in the negative x panel, there are nearly five times as many electron (generated only in the first 0.5 cm of water) that are travelling into the panel than in the positive x panel. Furthermore, there are roughly nine times as many electrons (generated in the water past the first 0.5 cm of the panel) travelling out of the panel in the positive x panel than in the negative. Overall, this indicates that the Lorentz force pushes electrons deeper into the phantom in the negative x panel and out of the phantom in the positive x panel. Electrons that exit the surface of the positive panel will curve, return to the water surface, and produce a higher surface dose as compared to the negative x panel and the 0 T simulations as seen in Fig. 5. The dose averaged over a 1 cm region in the MC results for the surface, 1.4, 2.7, and 4.0 mm depths is 2.21%, 1.71%, 1.45%, and 1.31%, respectively, for the positive x panel, and 0.57%, 0.91%, 1.06%, and 1.15% for the negative x panel (absolute uncertainty of 0.01%). For the experimental results at the same depths, the doses are 2.4%, 1.6%, 1.3%, and 1.1%, for the positive x panel, and 0.6%, 1.0%, 1.3%, and 1.1%, for the negative x panel (absolute uncertainty of about 0.2%). The MC and experimental results are all within two standard deviations of each other.

**Figure 6:**
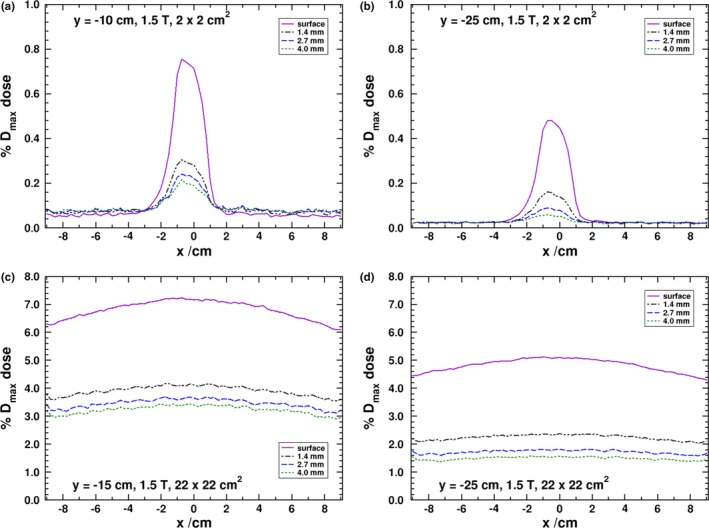
MC derived dose profiles at the surface and various depths in the negative y panel as shown in Fig. 2 with for a field size of either 2 cm × 2 cm, (a) 10 cm, or (b) 25 cm panel to isocenter distance or 22 cm × 22 cm, (c) 15 cm, or (d) 25 cm panel to isocenter distance. [Color figure can be viewed at http://www.wileyonlinelibrary.com/]

In Fig. 6, profiles for a field size of 2 cm × 2 cm, for a panel to isocenter distance of either 10 or 25 cm, and 22 cm × 22 cm, for panel to isocenter distances of 15 and 25 cm, are given. For the 2 cm × 2 cm field size, at 15 cm panel to isocenter distance, the peak doses are 0.61%, 0.21%, 0.13%, and 0.10% for the surface, 1.4, 2.7, and 4.0 mm depths (absolute uncertainty of 0.01%). The number of SCEs is proportional to the photon fluence, and the effect is as such directly proportional to the field size. The proportionality is fairly linear, as observed by Hackett et al.^17^, in the relation between the maximum dose and the side of the square of the field size. The smaller field size is 20% of the side length of the 10 cm × 10 cm field, and has a dose, averaged over 1 cm, of about 18% that of the 10 cm × 10 cm one at a panel to isocenter distance of 15 cm. For the same 15 cm distance, the larger field size is 220% longer and has about a 230% larger dose than the 10 cm × 10 cm field.

### 3.B. Changing distance from isocenter

**Figure 7:**
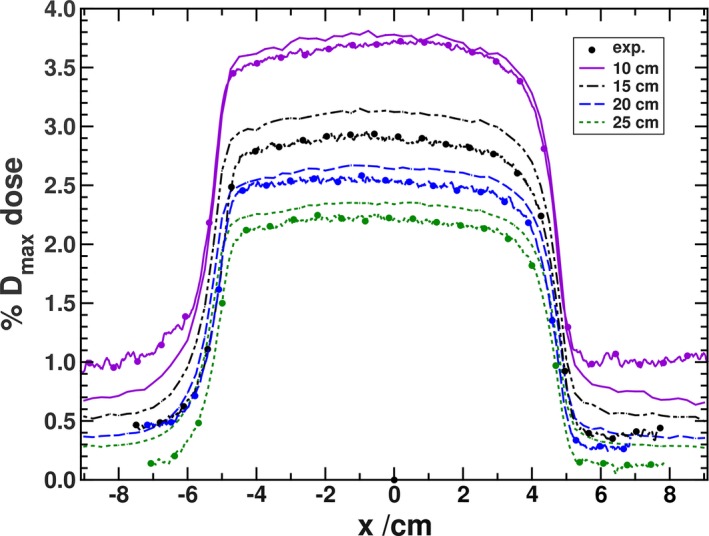
Surface dose profiles for the negative y panel in a 1.5 T magnetic field with the panel positioned at four distances away from isocenter. The solid circle curve represents the Hackett et al. experiments, except for the purple curves which are the updated experiments. [Color figure can be viewed at http://www.wileyonlinelibrary.com/]

Hackett et al. also produced surface dose profiles with the negative y panel positioned at 15, 20, and 25 cm away from isocenter. In Fig. 7, those results are reproduced using MC simulations. The solid purple curve in the figure is the same result as in Fig. 3(b) and the corresponding purple curve with the solid circles are the experimental result from Fig. 4(b). The Hackett et al. experimental results are the remaining solid circle curves. As the panel is moved further away, the electrons continue to travel along the magnetic field lines, while losing some of their energy and undergo additional scattering in the air. As such the overall shape of the profile remains the same at larger distances, but the magnitude of the surface dose is reduced. The surface doses, averaged in a 1 cm region centered on x = 0 cm are 3.12%, 2.65%, and 2.35% for the 15, 20, and 25 cm simulations, respectively (±0.01% uncertainty). These results match well to the previously measured values of about 2.9%, 2.5%, and 2.2% (± 0.2% uncertainty) for the same respective panel positions. Although the comparison at further panel positions is made with the measurements from Hackett et al.^17^, the reasonable agreement shown in the previous section between the MC and the updated experimental results could suggest that, at further panel positions, there is a reduced dependence on the details of the beam model.

**Figure 8:**
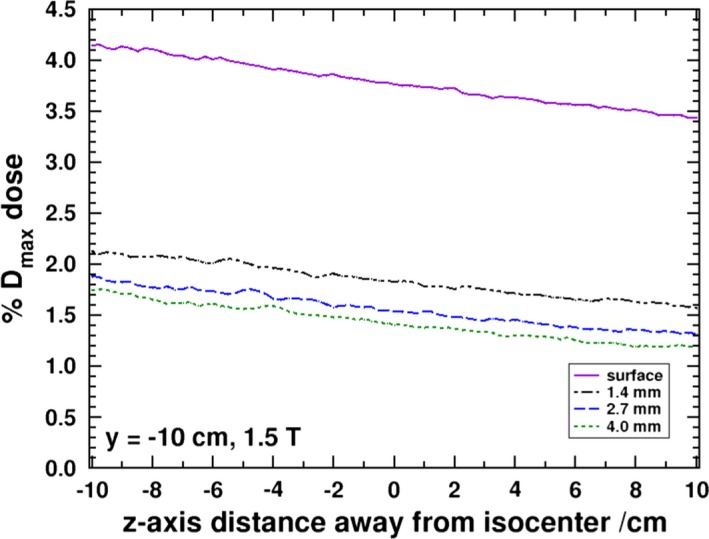
MC derived dose profiles along the z‐axis at the surface and various depths in the negative y panel. [Color figure can be viewed at http://www.wileyonlinelibrary.com/]

In Fig. 8, the dose profile along the z‐axis is provided for the four simulated depths in the negative y panel. There is a steady drop in the surface dose from about 4.1% to 3.4% along the 20 cm distance. This is equivalent to a roughly 17% drop in dose over this z‐axis distance, as predicted by MC, which is less than a reduction of about 24% if the inverse square law (ISL) was to be applied between an SSD of 133.5 and 153.5 cm. Part of the effect in the reduction would come from the attenuation of photons in the air (small component). Another contribution could potentially be the divergence of the beam along the y‐axis which would only lead to a change in the location production and not a substantial change in the photon fluence projected onto the negative y panel with increasing z‐axis depth. However, the beam also diverges along the x‐axis and the projection of the photon fluence, which is directly related to the electron fluence, along that direction onto the panel is inversely proportional to the z‐axis distance from the source. An inverse proportionality to the z‐axis distance would predict a 13% dose difference along the 20 cm long region. However, in addition to the SCE contribution, there is the photon scatter dose component which would still follow the ISL. It can be seen that, deeper into the panel, as the dose due to the SCEs become less important, the change in the dose along the profile is much closer to the ISL. For example, at 2.7 mm depth, the dose drops by about 26% along the 20 cm distance.

### 3.C. Out‐of‐field doses for 0.35 and 0 T

**Figure 9:**
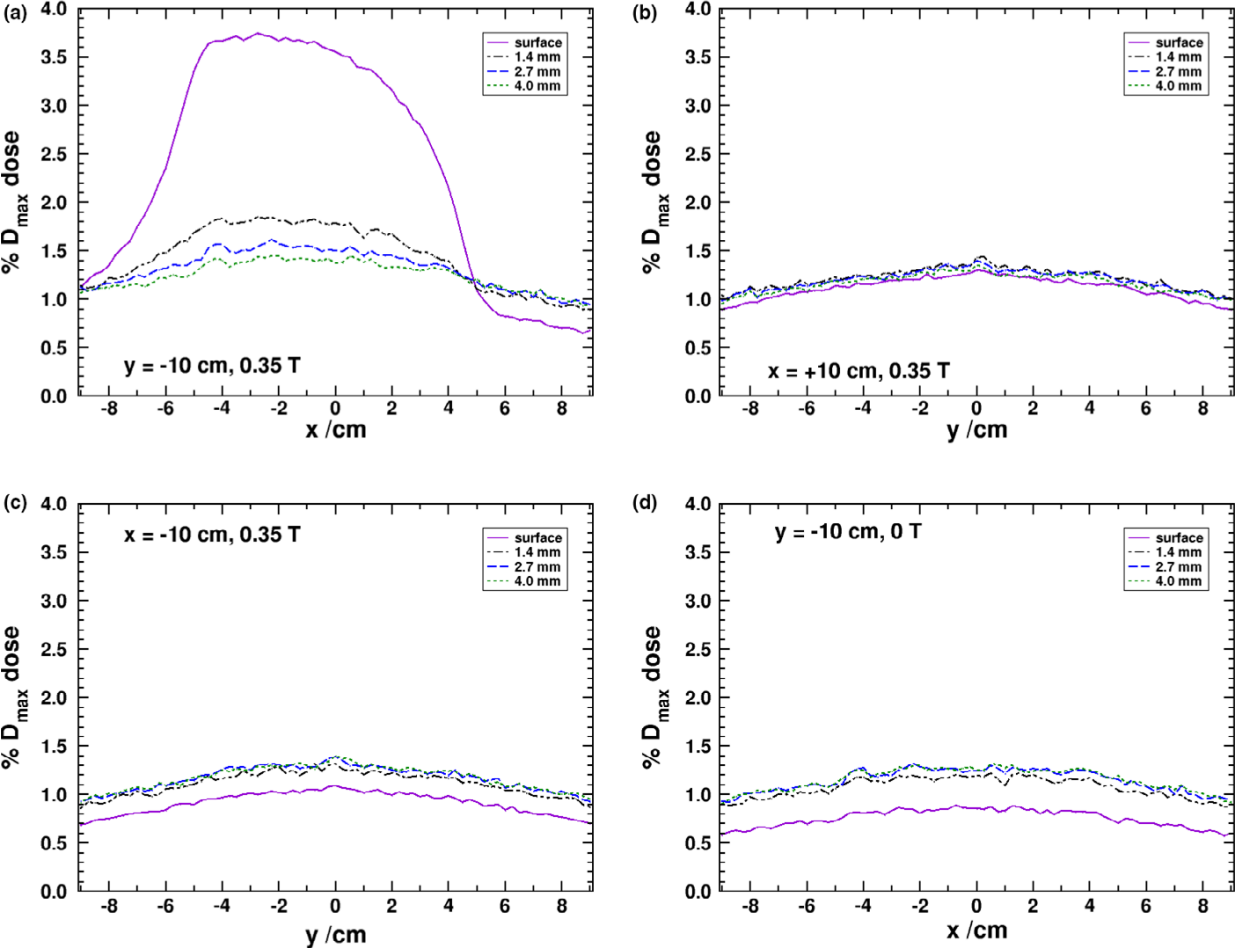
Dose profiles at the surface and various depths for 0.35 T for the negative y panel (a), for the negative (b) and positive (c) x panels, and for 0 T for the negative y panel (d). [Color figure can be viewed at http://www.wileyonlinelibrary.com/]

In Fig. 9, the dose profiles for 0.35 and 0 T are provided. As the y panels produce symmetric results, only the negative y and both x calculations are presented for 0.35 T. In the absence of a magnetic field, all four panels produced the same results, and because of this, only the negative y data are presented.

In Fig. 9(a), the effect of the magnetic field on the curvature of the electrons toward the negative x‐axis is very apparent. This is due to the gyroradius in the 0.35 T field being much larger than that of the 1.5 T magnetic field, and although the electrons still stream along the magnetic field, they are less localized to the primary beam profile. The dose in a 1 cm region centered on x = −3 cm is about 3.45 ± 0.17%. Although to a lesser degree, due to the much lower magnetic field, there is still a difference between the surface doses of the negative and positive x panels seen in Figs. 9(c) and 9(b). The dose in a 1 cm region centered on x = 0 cm is 1.27 ± 0.17% for both the positive and negative x panels.

The 0 T results in Fig. 9(d) are obtained using all of the photons and electrons from the phase space file. The inclusion of the electrons from the phase space file produced little impact on the overall dose distributions shown in the figures and implies that the photons are the dominant source of dose contribution. The simulated maximum water dose without a magnetic field was found to be 2.3 ± 0.1% lower than with a 1.5 T magnetic field. This variation is consistent with the findings of O’Brien et al.^24^ The results in the figure show that the scatter contribution 5 cm away from the field edge (y = −10 cm) for a 10 × 10 ${\text{cm}}^{2}$ beam in a 1 cm region centered on x = 0 cm is 0.86 ± 0.13% of the deliverable dose of the beam. Cozzi et al.^25^ measured, in a large water phantom with the surface perpendicular to the direction of the incoming photon beam, a dose of 1.5% at a depth of 0.15 cm and located 5 cm away from the field edge of a 10 × 10 ${\text{cm}}^{2}$ Varian 6 MV field. This dose measurement was expressed as a fraction of the dose delivered by the beam along the central axis in the water phantom in a depth of 5 cm. Here, the dose is roughly 85% of the maximum dose for the beam used in that study, and the dose measured 5 cm outside the field edge and normalized to the maximum is about 1.28% of the maximum dose. This is comparable to the values shown in Fig. 9(d), especially when considering the absence of additional scatter contributions due to a larger water phantom in the path of the photon beam.

### 3.D. Monte Carlo parameter sensitivity

An important component of MC calculations is the appropriate selection of simulation parameters. The simulations in this work test the capabilities of the code to correctly perform electron transport and dose calculations in magnetic fields at fairly large distances, as compared the ion chamber use cases in which the magnetic field transport had been previously used. Even in the absence of a magnetic field, the selection of simulation parameters can significantly impact the results, and here, variations due to these parameters are explored in order to guide this work and future studies.

**Figure 10:**
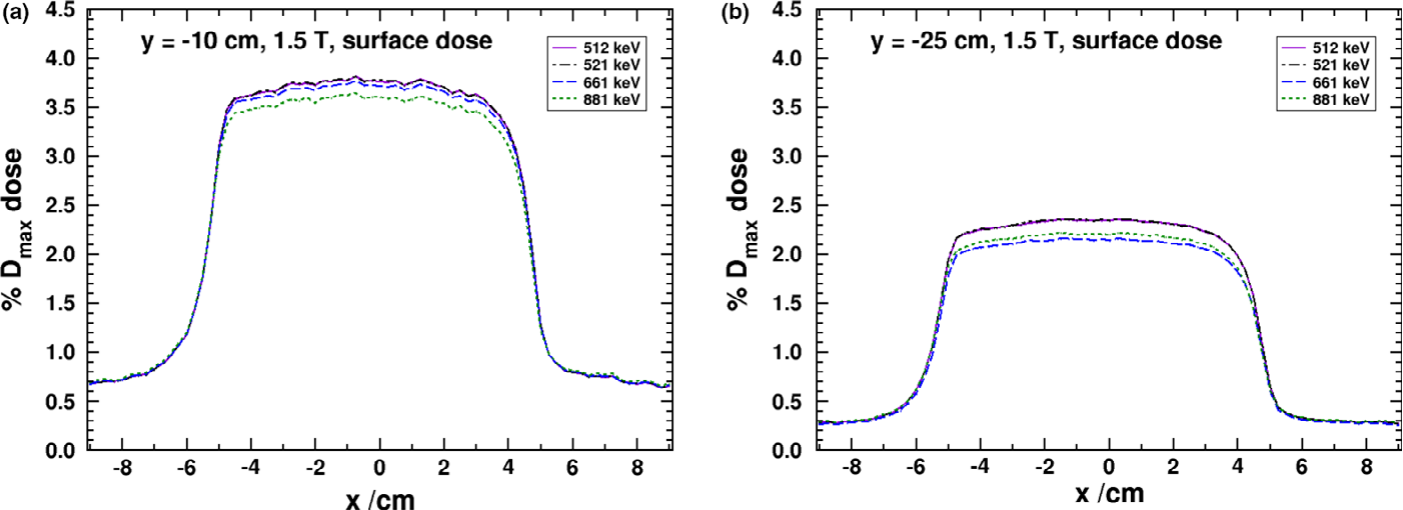
MC derived dose profiles at the surface and various depths in the negative y panel for various ECUT and AE values at a panel to isocenter distance of (a) 10 cm and (b) 25 cm. [Color figure can be viewed at http://www.wileyonlinelibrary.com/]

The electron total energy cutoff value, ECUT, in EGSnrc is a parameter which can impact simulation efficiency and dramatically influence the dose calculation if chosen incorrectly. In Fig. 10, dose profiles, for panel to isocenter distances of 10 and 25 cm, are shown with ECUT values of 512, 521, 661, and 881 keV. In each of the simulations AE, the lower energy threshold for electron generation is set to be equal to ECUT. For both distances, increasing ECUT to 521 keV produces no impact on the dose calculation and demonstrates the stability of the calculation. However, increasing ECUT to 661 keV produces a 1.2% and 8.4% drop in the central profile dose to the 10 and 25 cm simulations, respectively. Further, an 881 keV ECUT results in a 4.4% and 5.9% lower dose from the 512 keV ECUT simulations for the same respective distances. The reduction in dose is due to electron being prematurely terminated in the air prior to reaching the surface of the panel. The 25 cm panel position simulation is more sensitive to ECUT because electron lose energy as they travel through the air and this leads to more electron being terminated before reaching the panel. The dose in the 25 cm profile increases when using an 881 keV cutoff as compared to the 661 keV one due to a larger number of electron being stopped within the surface scoring layer in the water panel. The results for the deeper profiles are less sensitive to the selection of ECUT. Based on these results, the 521 keV ECUT value was chosen for all simulations as providing improved efficiency over the 512 keV calculations while not compromising the results. The x‐axis panel simulations were less sensitive to variations in ECUT, and only at 881 keV was a difference from the 512 keV results observed (ECUT values of 512, 521, 661, and 881 keV were tested).

As discussed in Malkov and Rogers,^22^ the step size in electron transport in magnetic fields needs to be correctly controlled to ensure that the magnetic field transport algorithm does not introduce errors to the simulations. In that work, it was recommended that EM ESTEPE, the parameter which limits the step size to ensure that the change in the direction of motion induced by the magnetic field is less than the set fractional value, should be set to 0.2. Recently, Lee et al.^26^ have found that the EGSnrc enhanced electric and magnetic field macros,^22^ which are used in this study, achieved the best accuracy in ion chamber Fano tests when compared to the magnetic field transport of PENELOPE, Geant4, and MCNP6. To evaluate the importance of this parameters on the dose calculations in this current work, simulations with the negative y panel at 10 and 25 cm were performed using EM ESTEPE values of 0.01, 0.1, 0.2, and 0.4. No differences in dose were observed in those simulations, which further demonstrates the robustness of the magnetic field transport. It is expected that EM ESTEPE has little impact in these simulations, as the magnetic field transport algorithm excels in transport in low‐density media like air due to the use of an analytical transport algorithm for single scattering electron steps (which occur much more frequently in low‐density media).

**Figure 11:**
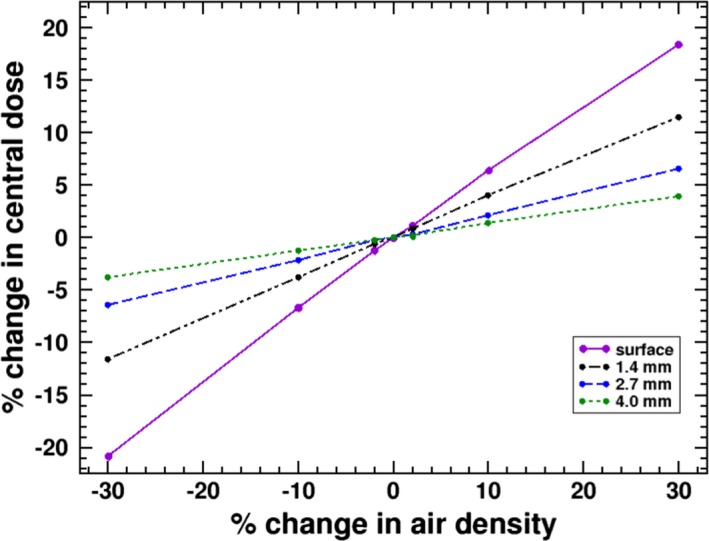
Percent change in the negative y panel doses in a 1 cm region for the four simulated depths and a panel to isocenter distance of 10 cm as a function percent change in simulated air density. [Color figure can be viewed at http://www.wileyonlinelibrary.com/]

In Fig. 11, the percent change in dose in a 1 cm region for the negative y‐panel simulations for the four simulated depth is given a function of percent change in the simulated air density. The reference air density used in all of the above simulations is 1.247e−3 g/cm${}^{3}$ which is for 20${}^{\circ }$C and 760 mmHg. A 30% change in density would require a substantial change in measurement conditions; however, a few percent pressure and temperature corrections can be common and, as seen in the figure, can produce several percent change in the measured doses. The effect is due to the proportionality of the number of photon interactions to the air density. Even a change in conditions to 22${}^{\circ }$C and 740 mmHg would produce a 3.3% drop in air density which would correspond to roughly 2% change in the measured surface dose and can introduce additional experimental uncertainty. The surface profile is most sensitive to this effect.

## 4 Conclusion

The surface dose enhancement to regions perpendicular to the magnetic field due to SCE has been confirmed in this study using the EGSnrc Monte Carlo code. The effect can be of the order of several percentage of the maximum deliverable dose by the photon beam and appears at 0.35 and 1.5 T magnetic field strengths. The spiralling contaminant electron (SCE) dose contribution is apparent in the first few millimeters of material. Furthermore, the simulations without a magnetic field demonstrated that the scatter dose contribution outside the main radiation field is near a percent at the surface of the water panel located 5 cm away from the field edge.

In the context of clinical treatments, these calculations have been performed for a single beam and the results are expressed as a percentage of the maximum deliverable dose by that beam in a water phantom, and as a percentage of the prescription dose, the results could be quite different. Furthermore, the MR‐linac delivers beams from multiple gantry angles during a treatment, and several gantry orientations, particularly posterior–anterior beams, will have a minimal SCE dose contribution. Park et al.^27^ observed recently isodose contours of as much as 15% of the prescription dose streaming away from the treatment area and in the direction of the magnetic field. Considering that the air‐generated SCE dose contribution is on the order of about 5% as shown by Hackett et al., this would indicate that further work is required into investigating the contribution of in‐patient generated electron to out‐of‐field doses.
